# Tissue‐specific deletion of mouse basolateral uniporter LAT4 (Slc43a2) reveals its crucial role in small intestine and kidney amino acid transport

**DOI:** 10.1113/JP280234

**Published:** 2020-09-16

**Authors:** Anuradha Rajendran, Nadège Poncet, Lalita Oparija‐Rogenmozere, Brigitte Herzog, François Verrey

**Affiliations:** ^1^ Institute of Physiology University of Zurich Zurich Switzerland; ^2^ NCCR Kidney. CH University of Zurich Zurich Switzerland

**Keywords:** amino acid homeostasis, amino acid transport, basolateral membrane, epithelium, kidney proximal tubule, knockout models, small intestine

## Abstract

**Key points:**

- LAT4 is a broadly expressed uniporter selective for essential branched chain amino acids, methionine and phenylalanine, which are involved in epithelial transport.
- Its global deletion leads to an early malnutrition‐like phenotype and death within 10 days after birth.
- Here, we tested the impact of deleting LAT4 selectively in the mouse intestine. This affected slightly the absorption of amino acids (AAs) and delayed gastrointestinal motility; however, it had no major phenotypic effect, even when combined with aromatic AA uniporter TAT1 knockout (KO).
- Conversely, kidney tubule‐selective deletion of LAT4 led to a substantial aminoaciduria that strongly increased under a high protein diet. Combining a partial tubular LAT4 deletion with TAT1 KO implicated their synergistic action on AA reabsorption.
- These results show that LAT4 plays an important role for kidney AA reabsorption, but that its functional role in intestinal AA absorption is largely dispensable.

**Abstract:**

Amino acid (AA) transporter LAT4 (Slc43a2) functions as facilitated diffusion uniporter for essential neutral AAs and is highly expressed at the basolateral membrane of small intestine (SI) and kidney tubule epithelia. Previously, we showed that LAT4 global knockout (KO) mice were born at the expected Mendelian ratio but died within 10 days. Their failure to gain weight and a severe malnutrition‐like phenotype contrasted with apparently normal feeding, suggesting a severe intestinal AA absorption defect. In the present study, using conditional global and tissue‐specific LAT4 KO mouse models, we nullified this hypothesis, demonstrating that the selective lack of intestinal LAT4 does not impair postnatal development, although it leads to an absorption defect accompanied by delayed gastrointestinal motility. Kidney tubule‐specific LAT4 KO led to a substantial aminoaciduria as a result of a reabsorption defect of AAs transported by LAT4 and of other AAs that are substrates of the antiporter LAT2, demonstrating, *in vivo*, the functional co‐operation of these two transporters. The major role played by basolateral uniporters in the kidney was further supported by the observation that, in mice lacking TAT1, another neutral AA uniporter, a partial LAT4 KO led to a synergistic increase of urinary AA loss. Surprisingly in the SI, the same combined KO induced no major effect, suggesting yet unknown compensatory mechanisms. Taken together, the lethal malnutrition‐like phenotype observed previously in LAT4 global KO pups is suggested to be the consequence of a combinatorial effect of LAT4 deletion in the SI, kidney and presumably other tissues.

## Key points

- LAT4 is a broadly expressed uniporter selective for essential branched chain amino acids, methionine and phenylalanine, which are involved in epithelial transport.
- Its global deletion leads to an early malnutrition‐like phenotype and death within 10 days after birth.
- Here, we tested the impact of deleting LAT4 selectively in the mouse intestine. This affected slightly the absorption of amino acids (AAs) and delayed gastrointestinal motility; however, it had no major phenotypic effect, even when combined with aromatic AA uniporter TAT1 knockout (KO).
- Conversely, kidney tubule‐selective deletion of LAT4 led to a substantial aminoaciduria that strongly increased under a high protein diet. Combining a partial tubular LAT4 deletion with TAT1 KO implicated their synergistic action on AA reabsorption.
- These results show that LAT4 plays an important role for kidney AA reabsorption, but that its functional role in intestinal AA absorption is largely dispensable.

## Introduction

Proteins and in extension amino acids (AAs) are key macronutrients of our everyday diet. AAs are crucial metabolites that are not only used as building blocks of proteins, but also as substrates for energy metabolism, precursors of biologically important molecules and neurotransmitters, as well as for many other functions (Makrides *et al*. 2014). Unlike the other two macronutrients (carbohydrates and fats), excess protein intake does not lead to their storage but, instead, mostly to their degradation (Arany & Neinast 2018). Hence, studies involving AA uptake (transport), utilization (metabolism) and disposal are of crucial importance to advance our knowledge on human nutrition. Mutations in genes involved in these pathways lead to numerous hereditary diseases, such as cystinuria (mutated Cys and cationic AA transporter b^0,+^‐rBAT), phenylketonuria (errors in Phe metabolism), maple syrup urine disease [defect in BCAAs (breakdown of branched chain amino acids)] and many others that cause a variety of symptoms affecting appetite, growth, behaviour, etc., in humans (Chillarón *et al*. 2010; Aliu *et al*. 2018; Bröer & Fairweather 2018; Sandlers 2019). Additionally, AAs and AA transporters have long been implicated in the pathogenesis of several metabolic and age‐related disorders, such as obesity, diabetes and cancer (Felig *et al*. 1969; Arany & Neinast 2018; Kandasamy *et al*. 2018; Bott *et al*. 2019; Häfliger & Charles 2019; Javed & Fairweather 2019).

The optimal quantity and quality of dietary proteins and/or AAs for adult humans is still being debated, with recent evidence pointing towards improved metabolic health under protein and/or specific AA restriction (Green & Lamming 2019; Kitada *et al*. 2019). However, sufficient protein intake is crucial for metabolic homeostasis throughout life and, in particular, in growing children, especially during the embryonic and postnatal periods (Green & Lamming 2019). Accordingly, many AA transporters (AATs) with diverse kinetic properties and overlapping AA selectivity have been identified in the placenta and all other organs, tissues and cell types throughout development (Makrides *et al*. 2014). Ingested proteins are the major source of body AAs. They are digested into small peptides and single AAs that are absorbed mainly by the small intestine (SI) enterocytes (Bröer & Fairweather 2018). In addition, the kidney plays a major role in overall AA homeostasis by reabsorbing AAs across its proximal tubule (PT) epithelium, thereby preventing their loss in the urine (Makrides & 2014).

These transepithelial transports in the SI and kidney PT are considered to be essentially transcellular, thus requiring specific transport proteins (carriers) to cross the two lipid membranes of the polarized epithelial cell barrier. The luminal transport into the epithelial cells is mediated by active transporters, mostly by symporters (cotransporters), such as Na^+^ symporter B^0^AT1 (Slc6a19) for most neutral AAs, and the H^+^ symporter PEPT1 (Slc15A1) for di‐ and tripeptides. One of the active luminal AA transporters is an antiporter (exchanger), b^0,+^AT‐rBAT (Slc7a9‐Slc3a1), which was mentioned above in the context of cystinuria (Fei *et al*. 1994; Saito *et al*. 1996; Romeo *et al*. 2005; Makrides *et al*. 2014). The net efflux of the neutral AAs from the epithelial cells across the basolateral membrane is postulated to be mediated by co‐operating AA exchangers and uniporters. Although almost all neutral AAs can be transported out of the cells by the AA antiporters LAT2 and y^+^LAT1, these antiporters exchange AAs and thus do not lead to an overall net AA efflux on their own. The antiporters nonetheless function as net efflux pathway for most non‐essential AAs. In exchange for this efflux of non‐essential AAs, they take up essential AAs that can then directionally recycle out of the cell via parallel uniporters (facilitated diffusion pathways) (Ramadan *et al*. 2007; Verrey & System 2003; Vilches *et al*. 2018).

To date, TAT1 and LAT4 are the only uniporters for essential neutral AAs known to be expressed in the basolateral membranes of SI and kidney tubule epithelia (Ramadan *et al*. 2006a; Guetg *et al*. 2015). TAT1 is a well characterized T‐type aromatic AA uniporter that mediates facilitated diffusion of essential aromatic AAs phenylalanine, tryptophan and tyrosine with low symmetric affinities (3–7 mmol L^−1^) (Ramadan *et al*. 2006b, 2007). It is highly expressed in the basolateral membranes of the SI, kidney, colon, placenta, liver, stomach, skeletal muscle and testis (Kim *et al*. 2001; Ramadan *et al*. 2006a). When co‐expressed with LAT2‐4F2hc in the *Xenopus laevis* oocyte expression system, TAT1 was shown to functionally co‐operate with LAT2 and drive the net efflux of nonessential AAs such as glutamine (LAT2 substrates) across the membrane (Ramadan *et al*. 2007). A recent study also demonstrated, *in vivo*, the co‐operation of TAT1 with antiporter LAT2 and presumably also with y^+^LAT1, driving the net efflux of mostly non‐essential intracellular neutral AAs (Vilches *et al*. 2018). However, studies with constitutional KO mice for TAT1 showed that the lack of this uniporter did not block the basolateral transport of any AA across (re)absorbing epithelia and led only to a mild phenotype (Mariotta *et al*. 2012). It was proposed that the parallel antiporter LAT2 would mediate, as a result of its selectivity overlap with TAT1, the efflux of aromatic AAs and that this exchange‐transport was driven by AAs effluxed via the other essential AA uniporter LAT4 that was still present.

LAT4 (Slc43a2) is a facilitated diffusion uniporter originally identified by Bodoy *et al*. (2005). It functions as a uniporter for a small subset of essential neutral AAs, including the branched ones (leucine, isoleucine, valine), methionine and phenylalanine with a low apparent affinity for its substrates (5 mmol L^–1^ for Phe). LAT4 is highly expressed in various tissues such as the placenta, SI enterocytes, kidney epithelium and peripheral blood leukocytes, and, to a lesser extent, in the brain, white adipose tissue and heart (Bodoy *et al*. 2005). Within the SI, LAT4 localizes to the villi at the basolateral membrane of enterocytes. In the kidney, it is expressed not only in epithelial cells of the PT, but also in the thick ascending limb and collecting duct, as well as in the distal tubule to a minor extent (Bodoy *et al*. 2005; Guetg *et al*. 2015).

To characterize the role of LAT4, our group originally generated a constitutional *Slc43a2* (Lat4) KO mouse model (Guetg *et al*. 2015). LAT4^−/−^ pups were born at a normal Mendelian ratio but displayed a slight intrauterine growth retardation (∼10%). Analysis of the amniotic fluid showed a strong decrease in AA levels that was not limited to LAT4 substrates, indicating a broader defect at the level of placental AA transport. After birth, these mice did not grow normally and displayed a starvation‐like phenotype with increased plasma acylcarnitines, reduced plasma non‐essential AA levels and low fasting plasma glucose levels. Although they appeared to feed normally as suggested by the milk‐spot in their belly, they did not gain much weight and died before day 10 (Guetg *et al*. 2015). We hypothesized that these mice suffered from a severe defect in the intestinal absorption. However, whether this intestinal defect was the main reason for the lack of growth of the pups could not be determined because they did not survive beyond the postnatal phase. This early postnatal death also made it difficult to investigate the impact of LAT4 defect on the function of other organs.

Thus, many questions remained and, in particular, it was not clear which role(s) of LAT4 were responsible for the postnatal malnutrition phenotype and death of the global KO mice. Was it a result of the lack of LAT4 in the intestine and kidney and the consecutive defective AA (re)absorption? Or was it a result of the lack of LAT4 function in other tissues? And would a deletion of LAT4 induced only past the postnatal phase result in a lethal phenotype as well? To address these questions, we generated and studied a number of conditional LAT4 KO mouse models. Additionally, by knocking out both LAT4 and TAT1 in the SI and kidney, we addressed the question of whether together these two major AA uniporters expressed at the basolateral membrane might be a requisite for the basolateral efflux of AAs and thus AA transport across (re)absorbing epithelia.

## Methods

### Ethical approval

All animal experiments were carried out according to the Swiss animal welfare laws and approved by the Cantonal Veterinary Office of Zürich (references #ZH075/15; #ZH228/17 and #ZH206/18). The study was conducted in compliance with the ethical principles and standards of *The Journal of Physiology*.

### Animals

#### Generation of floxed LAT4 mice

Lat4 (Slc43a2) floxed mice, *LAT4^f/f^* were produced by PolyGene Transgenetics (Rümlang, Zürich, Switzerland). The targeting vector containing exon 5 of the *Slc43a2* gene floxed with LoxP sites and a FRT flanked neomycin cassette (allowing for positive selection of clones) was transfected into C57Bl/6N‐derived embryonic stem cells by electroporation. Positive clones containing the correct homologous recombination were further identified by PCR analysis and injected into blastocysts from grey C57Bl/6 mice, which were then transferred into CD‐1 foster mice. Resulting chimeric mice were mated with grey Flp‐deleter mice (C57Bl/6N derived) to remove the neomycin cassette.

#### Generation of LAT4 KO mouse models

**Figure 1:**
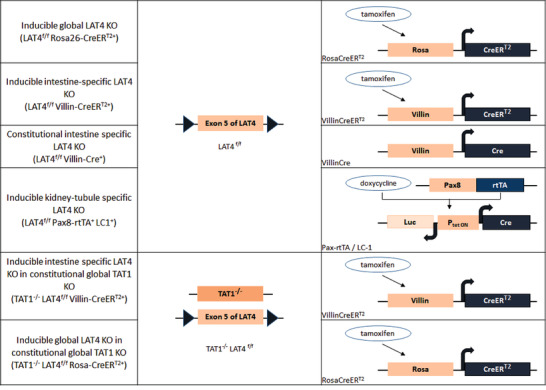
KO strategies for the mouse models Schematic representation of the KO strategies used for the different mouse models [Color figure can be viewed at wileyonlinelibrary.com]

Inducible global, intestine‐ and kidney tubule‐specific LAT4 KO mouse models were generated by breeding *LAT4^f/f^* mice with mice containing *Rosa26‐CreER^T2+^* (B6.129‐Gt(ROSA)26Sortm1(cre/ERT2)Tyj/J) (IMSR catalogue no. JAX:008463, RRID:IMSR_JAX:008463; The Jackson Laboratory, Bar Harbor, ME, USA) (Ventura *et al*. 2007), *Villin‐CreER^T2+^* (B6N.Cg‐Tg(Vil1‐cre/ERT2)23Syr/J) (IMSR catalogue no. JAX:020282, RRID: IMSR_JAX:020282; The Jackson Laboratory) (El Marjou *et al*. 2004) and *Pax8‐rtTA^+^/LC1^+^* (B6.Cg‐Tg(Pax8‐rtTA2S*M2)1Koes/J) (IMSR catalogue no. JAX:007176, RRID:IMSR_JAX:007176; The Jackson Laboratory) (Traykova‐Brauch *et al*. 2008) transgenes, respectively (Fig. 1). Constitutional intestine‐specific LAT4 KO mice were produced by breeding *LAT4^f/f^* mice with *Villin‐Cre^+^* mice [B6.Cg‐Tg(Vil1‐cre)997Gum/J] (IMSR catalogue no. JAX:004586, RRID:IMSR_JAX:004586; The Jackson Laboratory) (El Marjou *et al*. 2004). Transgenic *Pax8‐rtTA^+^/LC1^+^* and *Villin‐Cre^+^* mice were kind gifts from Dr N. Hernando, University of Zurich (Zurich, Switzerland).

#### Generation of LAT4 and TAT1 double KO (dKO) mouse models

Global and intestine‐specific LAT4 (inducible) and TAT1 (constitutional) dKO mouse models were generated by breeding TAT1 null (*TAT^−/−^*) mice (Mariotta *et al*. 2012) with *LAT4^f/f^ Rosa26‐CreER^T2+^* and *LAT4^f/f^ Villin‐CreER^T2+^* mice, respectively (Fig. 1).

All animals were bred in the LASC husbandry of the University of Zurich under standard conditions and had *ad libitum* access to water and a standard chow diet unless otherwise specified. The number of mice used in each animal experiment is indicated where appropriate. All experiments were initiated (induction of KO) in 8‐week‐old mice of both genders unless otherwise specified. We did not observe any gender‐specific differences in our results.

### Genotyping

Genomic DNA was isolated from ear biopsies by adding 20 μL of microLYSIS‐Plus buffer (catalogue no. 2MLP‐250; Gel company, San Francisco, CA, USA) followed by placing in a thermal cycler (SensoQuest Labcycler, Göttingen, Germany) in accordance with the manufacturer's instructions.

All genes were amplified using conventional PCR in the thermal cycler. The primers (Microsynth, Balgach, Switzerland) used in each reaction are summarized in Table 1.

**Table 1:** Primers used for genotyping the target alleles

Gene	Primer name	Primer sequence
Slc43a2 (LAT4)	Primer 1	CAG GTC CTT TCC TTC CAT TC
	Primer 2	ATG ACA GAT CCC GTC TTC TC
Rosa26‐CreER^T2^	Primer 1	AAA GTC GCT CTG AGT TGT TAT
	Primer 2	GGA GCG GGA GAA ATG GAT ATG
	Primer 3	CCT GAT CCT GGC AAT TTC G
Villin‐CreER^T2^	Primer 1	AAA GTC GCT CTG AGT TGT TAT
	Primer 2	GGA GCG GGA GAA ATG GAT ATG
Villin‐Cre	Primer 1	TCG CTG CAT TAC CGG TCG ATG C
	Primer 2	CCA TGA GTG AAC GAA CCT GGT CG
Pax8	Primer F	CCA TGT CTA GAC TGG ACA AGA
	Primer R	CTC CAG GCC ACA TAT GAT TAG
LC1	Primer F	TTA CAG ATG CAC ATA TCG AGG
	Primer R	TAA CCC AGT AGA TCC AGA GG
Slc16a10 (TAT1)	Primer A	GGG ACC CTC GGA TGT CTC
	Primer C	GTC GGT GTT TGG CAT CCA GAA CGC CAA A
	Primer D	GTG TCC AGC ATG GAC ACG AAG AGC ACC TCG
	Primer E	GGC CAT GTT GTC ATC GTC CTT GGC CTT GA

### Induction of LAT4 KO

#### Tamoxifen treatment

Inducible global and intestine‐specific LAT4 KOs (*LAT4^f/f^ Rosa26‐CreER^T2+^* and *LAT4^f/f^ Villin‐CreER^T2+^*) and control littermates (*LAT4^f/f^ Rosa26‐CreER^T2‐^* and *LAT4^f/f^ Villin‐CreER^T2−^*) were treated similarly with tamoxifen (catalogue no. T5648; Sigma‐Aldrich, Buchs, Switzerland) [Stock: 50 mg mL^–1^ prepared by dissolving tamoxifen in corn oil (catalogue no. C8267; Sigma‐Aldrich) containing 5% ethanol, giving a dose of 2 mg or 3 mg per 25 g of body weight per day for five consecutive days via i.p. injections to 3–4‐week‐ or 8‐week‐old mice, respectively. These mice were then given a recovery time (waiting period for DNA recombination and mRNA and protein degradation) of at least 10 days (exact time for each experiment is specified in the results section) after the first tamoxifen injection before performing any experiments. LAT4 KO in the LAT4 TAT1 dKO mouse models (*TAT1^−/−^ LAT4^f/f^ Rosa26‐CreER^T2+^* and *TAT1^−/−^ LAT4^f/f^ Villin‐CreER^T2+^*) was also induced by similar tamoxifen treatment.

#### Doxycycline treatment

Kidney tubule‐specific LAT4 KO was induced in 3–4‐week‐ or 8‐week‐old mice (*LAT4^f/f^ Pax8‐rtTA^+^/LC1^+^*) by administering 2 mg mL^–1^ doxycycline hyclate (catalogue no. 44577; Sigma‐Aldrich) in 2% sucrose via drinking water for 10 days followed by a recovery period of at least 4 days (the exact time for each experiment is specified in the Results) with normal drinking water. Control littermates, which are either *LAT4^f/f^ Pax8‐rtTA^−^/LC1^+^* or *LAT4^f/f^ Pax8‐rtTA^+^/LC1^−^*, were treated similarly with doxycycline. As a result of its unstable nature, doxycycline in sucrose was freshly made and replaced once every 2–3 days.

### Diets

Unless specified, all animals were fed a standard laboratory chow diet containing 18% casein (Kliba Nafag, Kaiseraugst, Switzerland). In an attempt to challenge the transport capacity of the amino acid transporters investigated, we also used diets with varying protein amounts: a low protein diet (LPD, 8% casein), a high protein diet (HPD, 40% casein) and a normal protein diet (NPD, 18% casein), which are all modified standard AIN93G diets (Reeves *et al*. 1993) maintained isocaloric with carbohydrate substitution (Kliba Nafag). All modified diets were also irradiated to match the hygiene regulations of our animal facility. All animals were acclimatized to the new diet for at least 1 week before performing any experiments.

### Time restricted feeding (TRF)

To identify potential compensation by other known basolateral amino acid transporters, we exposed mice to more stringent conditions, such as TRF only for 8 h during their active phase, a regime that has been shown previously to synchronize the expression of nutrient responsive genes. For experiments involving TRF of the inducible global and intestine‐specific LAT4 KO and control mice, 8‐week‐old animals were housed in climate chambers that allow for a customized time setting such that the onset of dark period, Zeitgeber time (ZT)12 was set to 09.00 h to allow manual addition/removal of food for 8 h in the active phase (of mice and the working personnel). Temperature and humidity of the chamber was matched to the standard conditions present in the animal housing. All animals were acclimatized to this inverted dark/light cycle for 1 week, followed by 1 week of acclimatization to the new diet (LPD/NPD/HPD), if any. Animals were then time restrictedly fed only for 8 h during their active phase from ZT12 to ZT20 for at least 1 week before the KO was induced (as described above). Mice were under TRF until they were killed (as described below for organ isolation) after a recovery period of 10–12 days post first tamoxifen injection. TRF was performed by manually changing the animals from a cage with no food to a cage containing food at ZT12 and vice versa at ZT20. All animals had *ad libitum* access to drinking water. None of the above‐mentioned changes (of housing in climate chambers or inverting dark/light cycle or subjecting to different diets) led to any changes in food intake or growth of mice.

### Blood glucose measurements

Mice that were time restrictedly fed for 2 weeks and hence fasted for 16 h before ZT12 were used to test the fasting blood glucose levels at 10–11 weeks of age. Blood samples were collected by cutting the tip of the tail and blood glucose levels were measured with reactive test stripes Accu‐Check (Roche Diagnostics, Mannheim, Germany).

### Body composition analysis

The body composition for each mouse was analysed using an EchoMRI body composition analyser (EchoMRI LLC, Houston, TX, USA). The animal was placed in a plastic holder (no sedation or special preparation), the holder was inserted into a tubular space in the EchoMRI system and scanning performed. Whole body fat, lean, free water and total water masses in live mice were delivered as numeric values.

### Organ isolation

Mice were anaesthetized with Attane Isolfurane (catalogue no. NDC 66794‐014‐10; Primal Critical Care Inc., Bethlehem, PA, USA) and then killed by cervical dislocation, which was immediately followed by cardiac incision and blood collection in heparin coated syringes. Blood plasma was separated by centrifuging the blood at 8000 *g* for 5 min at 4°C. All plasma and urine samples were stored at ‐20°C until further analysis. Full SI, kidney, liver, adrenal glands, pancreas, and white and brown adipose tissue were then isolated and either snap frozen in liquid nitrogen or transferred to 4% paraformaldehyde (PFA) and processed further for immunofluorescence (IF). Harvested SI was further cut into the designated segments: duodenum, jejunum and ileum, which were further divided in parts that were either inverted and scraped (using a scalpel) to collect the mucosa (epithelium) or cut open and fixed flat for 1 h in 4% PFA at 4°C, followed by ‘Swiss‐rolling’ (SR) (Moolenbeek & Ruitenberg 1981) and processing further for IF (see below). The SI was divided as: first 5 cm duodenum for scraping, next 5 cm for duodenal SR IF, the next 5 cm for Jejunum SR IF, the last 3 cm ileum for scraping, the second last 4 cm for ileum SR IF, and the remaining intestine that corresponds largely to jejunum was scraped. For western blotting (WB), to minimize proteolytic cleavage of LAT4 in duodenum and jejunum (Oparija *et al*. 2019), we isolated intestinal villi fractions (instead of using scraped mucosa) as described in our recent study (Oparija‐Rogenmozere *et al*. 2020). In the case of pups, killing was by decapitation. The SI was harvested and divided into several parts and intact small intestinal segments were either snap frozen or Swiss‐rolled for IF.

### Antibodies

The primary antibodies used were: rabbit anti‐LAT4, guinea pig anti‐B^0^AT1 (Guetg *et al*. 2015), rabbit anti‐LAT2 (Vilches *et al*. 2018) and mouse anti‐ß actin (Oparija *et al*. 2019) as described previously. The secondary antibodies used include: Alexa Fluor 594 anti‐rabbit (catalogue no. ab96921, lot no. GR170389‐3, RRID:AB_10680407; Abcam via Life Technologies, Carlsbad, CA, USA) and Alexa Fluor 488 anti‐guinea pig (Abcam, Cambridge, UK) for IF. Four, 6‐diamidino‐2‐phenylindole (DAPI) (catalogue no. D3571, lot no. 778144, RRID:AB_2307445; Thermo Fisher Scientific; Invitrogen, Carlsbad, CA, USA) staining was performed to visualize cell nuclei. For WB, anti‐rabbit IgG HRP conjugate (catalogue no. W4011, lot no. 0000340771; RRID:AB_430833) and anti‐mouse IgG (H+L) AP conjugate (catalogue no. S3721, lot no. 0000312817; RRID:AB_430871) were used.

### Sample fixation and IF

Organs and intestinal Swiss‐rolls were fixed in 4% PFA overnight, washed in PBS for 3 h, incubated in 15% sucrose for 5 h followed by incubating in 30% sucrose overnight, all at 4°C. All organs were then incubated in a 1:1 mixture of 30% sucrose and OCT (catalogue no. 81‐0771‐00; Biosystems, Muttenz, Switzerland) for 1 h at room temperature (RT). Organs were then embedded in OCT on dry ice.

For tissue sections, 5 μm cryosections were cut on SuperfrostPlus Menzel slides (catalogue no. J1800AMNZ; Gerhard Menzel GmbH, Braunschweig, Germany). For investigating LAT4 co‐localization with different kidney tubule segments, consecutive sections of 3 μm thickness were used. Slides were then briefly washed in PBS followed by epitope retrieval in 10 mM sodium citrate, pH 6.0, for 10 min at 98°C in a microwave (Histos 3; Milestone, Shelton, CT, USA). Slides were again washed in PBS 2× for 5 min each and washed in hypertonic PBS for 5 min. All sections were then incubated in blocking solution (2 % bovine serum albumin, 0.04% Triton X‐100 in PBS, pH 7.4) for 1 h at RT. Primary antibodies were also diluted in the blocking solution (LAT4, dilution 1:1000; B^0^AT1, dilution 1:250) and incubated overnight at 4°C. Sections were again washed in PBS and hypertonic PBS as described above and incubated with secondary antibodies (Alexa Fluor 594 anti‐rabbit and Alexa Fluor 568 anti‐guinea‐pig) and DAPI diluted 1:500 in blocking solution for 1 h at RT. Sections were then washed again and mounted using Dako Fluorescent Mounting Media (catalogue no. GM304; Agilent Technologies, Santa Clara, CA, USA) and imaged using a fluorescence light microscope (Eclipse TE 300; Nikon, Tokyo, Japan).

### WB

Scraped intestinal mucosa, villi fractions or kidney (cortex separated from medulla by cutting the kidney outline using a scalpel) were homogenized in 400 μL of mannitol resuspension buffer (200 mm D‐mannitol, 80 mm Hepes, 41 mm KOH, pH 7.5) supplemented with Protease inhibitor (catalogue no. P8340; Sigma‐Aldrich) and phosphatase inhibitor (catalogue no. 100567; Active Motif, La Hulpe, Belgium) cocktails. Homogenization was performed at 6000 rpm for 2 × 30 s with MagNa Lyser Green Beads (catalogue no. 03358941001; Roche Diagnostics) using the Precellys24 homogenizer (Precellys24; Bertin Instruments, Montigny‐le‐Bretonneux, France). The homogenate was then centrifuged at 2000 *g* for 15 min at 4°C and the resulting supernatant was then ultracentrifuged at 103,000 *g* (41000 rpm with rotor RP45A; Sorvall; Thermo Fisher Scientific, Waltham, MA, USA) to pellet down the membrane fraction. The resulting pellet was resuspended in 50 μL of mannitol resuspension buffer using a tip sonicator (Labsonic 1510; Bender & Hobein, Zurich, Switzerland). Protein concentration in the samples was determined using Pierce BCA Protein Assay (catalogue no. 23228; Thermo Fisher Scientific, Rockford, IL, USA) in accordance with the manufacturer's instructions. Protein samples in chosen concentrations were then diluted either in 4× Laemmli buffer containing 10% β‐mercaptoethanol and incubated for 20 min at RT (for LAT4) or in 400 mm dithiothreitol in Laemmli buffer and heated for 5 min at 95°C (for LAT2). Samples were then loaded to 1 mm 10% polyacrylamide gel and separated by electrophoresis followed by wet transfer on to a polyvinylidene fluoride membrane (catalogue no. IPVH00010; Immobilion‐P; Merck Millipore, Burlington, MA, USA). Membranes were then transferred to blocking solution [5% milk powder in Tris‐buffered saline containing 0.1% Tween‐20 (TBS‐Tween)] for 1 h at RT. Primary antibodies were also diluted in the blocking solution (anti‐LAT4, dilution 1:2000; anti‐LAT2, dilution 1:1000) and membranes were incubated overnight on a shaker at 4°C. Membranes were then washed in TBS‐Tween for 3 × 10 min and incubated for 1 h at RT in secondary antibodies also diluted 1:5000 in the blocking solution. β‐actin was used as loading control, and both primary and secondary antibodies were diluted 1:5000 in blocking solution and incubated 1 h at RT. Antibody binding was detected using Luminata Classico Western Chemiluminescent HRP substrate (catalogue no. WBLUC0100; Merck Millipore) or CDP‐Star substrate (catalogue no. NIF1229; GE Healthcare, Little Chalfont, UK). Blots were then imaged with a ImageQuant LAS 4000 camera (Fujifilm, Tokyo, Japan) and further densitometric analysis was performed using ImageJ (NIH, Bethedsa, MD, USA).

### Total RNA extraction and quantitative real‐time PCR

Total RNA was extracted using RNeasy Mini Kit (catalogue no. 74106; Qiagen, Hombrechtikon, Switzerland) in accordance with the manufacturer's instructions, with the exception that the frozen samples were lysed with 1 mL of Trizol (catalogue no. 15596026; Ambion Life Technologies, Carlsbad, CA, USA) (instead of RLT buffer) using MagNa Lyser Green Beads (catalogue no. 03358941001; Roche Applied Science, Basel, Switzerland) in a Precellys 24 homogenizer (6000 rpm, 2 × 30 s). The quality and quantity of the extracted RNA were assessed using a RNA 600 Nano kit (catalogue no. 5067‐1511; Agilent Technologies) and a Nanodrop ND‐1000 UV‐spectrophotometer (NanoDrop Technologies, Wilmington, DE, USA), respectively. Reverse transcription was then performed with qScript cDNA synthesis kit (catalogue no. 95047; Quantabio, Inc., Beverly, MA, USA) using 500 ng of RNA as template. For real‐time PCR, TaqMan Universal PCR Mastermix (catalogue no. 4304437; Applied Biosystems, Waltham, MA, USA), 75 ng cDNA and other reaction components were mixed in accordance with the manufacturer's instructions. The primers and probes (Microsynth, Balgach, Switzerland; except for LAT1 probe #34 from the Universal Probe Library, Roche) used in the study are provided in Table 2. The abundance of the target mRNAs (test RNA) was normalized relative to the 18S ribosomal RNA (18S rRNA), which was used as an internal standard in each reaction. All reactions were performed in triplicates and relative expression ratios were calculated as *R* = 2^−[Ct(test RNA)‐Ct(18S rRNA)]^, where Ct is the cycle number observed at the threshold for the tested mRNAs.

**Table 2:** Primers and probes used in the quantitative PCR analysis

Gene	Annotation	Accession number	Forward primer	Reverse primer	Probe
*Slc43a2*	*Lat4*	NM_173388	GCTGATTGCATATGGAGCAAGTAAC	CGAAGTGAACGTCATGCACAT	CTCTCTGTGCTCATCTTTATCGCCTTGGC
*Slc7a5*	*Lat1*	AB017189.1	TTTGCTTGGCTTCATCCAGAT	GGACAACTTCTGCTGCAGGTT	AAGGACATGGGACAAGGTGATGCGTC
*Slc7a8*	*Lat2*	NM 016972	TCCACGTTTGGTGGAGTCAAT	TGGATCATGGCCAACACACT	CTCCCTCTTCACCTCCTCCCGGCT
*Slc1a5*	*Asct2*	D85044	CATCCTGGAAGCAGTCAGCC	CACCTTCCACGTTGAGGACA	TGATCTTGGCCGTGGACTGGCTAGTG
*Slc7a7*	*y^+^Lat1*	NM 011405	AATTCCAGTAGCGGTTGCATT	GGAGGTGGCCTTCTCTCGAG	TTGCTTTGGTGGGCTCAACGCC
*Slc16a10*	*Tat1*	NM_028247.4	CGCCTACGGGGTGCTCTTC	ACTCACGATGGGGCAGCAG	CGAGCCCACCCACGCTGTCTTG
*Slc3a2*	*4F2hc*	NM_008577	GTTTTTGAATGCCACTGGCA	GTCCTGAGGAGCGTCTGAAA	ATGGTGCAGCTGGAGTGTGTCGCA
*Slc6a19*	*B^0^AT1*	NM_028878.3	GCCACTGAGCGCTTTGATG	GCCTCAAAGTTCTCTGAAGTCACA	ATGGGTTCGACCTGCCGGAGG
*Slc5a1*	*Sglt1*	NM_019810	GTTGGAGTCTACGCAACAGCAA	GGGCTTCTGTGTCTATTTCAATTGT	TCCTCCTCTCCTGCATCCAGGTCG
*Slc2a2*	*Glut2*	NM_031197.2	GGCCCTTGTCACAGGCATT	CCTGATTGCCCAGAATAAAGCT	TTATTAGTCAGATTGCTGGCC
*Slc38a1*	*Snat1*	AF184240	CGCGTGCACACCAAAGTATG	AGATTGGCAGGACGGACG	TACCAACCATCGCCTTCGCGTTTG
*Slc38a2*	*Snat2*	BC041108	CCAATGAGATCCGTGCAAAA	TGGACCCAATCCAGCACAAT	TCTGTGTTTTCTCCTGAGTGGCATAGTGGTG
*Slc38a3*	*Snat3*	AF159856	CGAATCATGCCCACTGACAA	AACCGCAGCGAAACAAAGG	AGCCTGCAAGATCCACCCCTAAAATCCT

### Functional analysis of LAT4 (and TAT1) mediated transport *in vivo*

Mice (8–12 weeks old) were food deprived for 11 h (22.00 h to 09.00 h the next day) before giving an oral gavage of an AA mix (10 μL g^–1^ mouse) containing all the proteinogenic AAs in 1 × PBS (pH 7.4) at a concentration 10× higher than the plasma AA concentration of wild‐type mice (Table 3) (Singer *et al*. 2012). In addition, the AA mix was supplemented with 3 μCi mL^–1 3^H‐ and 0.3 μCi mL^–1 14^C‐radiolabelled AA tracers and 60 mg mL^–1^ fluorescence labelled fluorescein isothiocyanate (FITC)‐dextran 4 kDa (catalogue no. 46944; Sigma‐Aldrich). Blood samples were collected 10 min before and at several time points [2, 5, 10, 20 (and 60) min] after the oral gavage from the tip of the tail into heparin coated capillaries. One hour (or 20 min) after the gavage, mice were killed as described above and blood was collected by cardiac puncture. The SI was divided into four equal parts and the intestinal tissue and its contents (washed with 1 mL of 1 × PBS pH 7.4 at room temperature) collected separately. Volume (10 μL g^–1^ body weight) and osmolality of the gavage fluid (∼350 mosmol kg^–1^) were the same as in previous experiments in which no deleterious effects had been observed (Singer *et al*. 2012).

**Table 3:** AA concentration in the AA mix [10× higher than in the plasma of wild‐type (WT) mice]

AA	Plasma WT (μm)	10 × plasma WT (μm)
Ala	689.38	6.89
Arg	75.02	0.75
Asn	89.62	0.90
Asp	31.94	0.32
Cys	30.00	0.30
Gln	953.09	9.53
Glu	110.82	1.11
Gly	338.57	3.39
His	67.75	0.68
Ile	105.95	1.06
Leu	183.72	1.84
Lys	582.61	5.83
Met	83.59	0.84
Phe	62.35	0.62
Pro	159.27	1.59
Ser	174.49	1.74
Thr	321.96	3.22
Trp	89.77	0.90
Tyr	79.30	0.79
Val	302.31	3.02

Isolated intestinal tissue and its flushed contents were then digested with 1 mL of Solvable (catalogue no. 6NE9100; PerkinElmer, Schwerzenbach, Switzerland) overnight at 50°C followed by destaining with 30% H_2_O_2_ (200 μL) for 1 h at RT on a shaker. Radioactivity in the blood plasma and the bleached intestinal tissue and contents were then measured by liquid scintillation counting in Ultima Gold solution (catalogue no. 6013329; PerkinElmer) using a ß‐counter (Packard Tri‐Carb 2900TR; PerkinElmer). The radiolabelled AA tracers used in the present study include: ^3^H‐Leu (catalogue no. ART 0840), ^3^H‐Mannitol (catalogue no. ART 0263); ^14^C‐Lys (catalogue no. ART 0243), ^14^C‐Trp (catalogue no. ARC 0254) and ^14^C‐Met (catalogue no. ARC 0271 (Hartmann Analytic GmbH, Braunschweig, Germany). The concentration of the AAs was calculated and the results are expressed as pmol mg^–1^ wet segment weight for intestinal tissue, as concentration (nmol μL^–1^) for luminal contents and as pmol μL^–1^ for plasma.

The appearance of FITC‐dextran in the blood was determined by measuring the fluorescence intensity (excitation 490/emission 530) of plasma collected at different time points using a plate reader (Infinite 200 PRO; Tecan Männedorf, Switzerland).

### Spot urine and plasma collection

Kidney tubule‐specific LAT4 KO mice (*LAT4^f/f^ Pax8‐rtTA^+^/LC1^+^*) were housed under standard laboratory conditions and had *ad libitum* access to food and water until 3 days before the urine and blood sample collection. Starting from 3 days before sample collection, animals were time restrictedly fed only for 16 h from ZT12 to ZT4 (18.00 h to 10.00 h the next day). On the day of sample collection, mice were restrained before feeding (18.00 h) in an attempt to make them empty the bladder. Two hours after feeding (20.00 h), mice were restrained on parafilm to collect spot urine followed by blood collection from the tail vein into heparin coated capillaries (Provet AG, Lyssach, Switzerland).

### Metabolic cage studies

In experiments involving metabolic cages, mice (*LAT4^f/f^ Pax8‐rtTA^+^/LC1^+^*, *TAT1^−/‐^ LAT4^f/f^ Rosa26‐CreER^T2+^ dKO)* were time restrictedly fed for 3 days as described above and were also acclimatized to the cages for ∼3 h during their inactive phase (from 13.00 h to 16.00 h). Two days after the training, mice were again transferred to the metabolic cages (at 18.00 h). Standard chow or a HPD was ground as a feed for the mice in metabolic cages. All animals had *ad libitum* access to water. Urine samples were then collected overnight (from 18.00 h until 10.00 h the next day) and water consumption was recorded. Mice were then anaesthetized with Attane Isolfurane (catalogue no. NDC 66794‐014‐10; Primal Critical Care Inc.) and killed by cardiac incision followed by blood collection using heparin coated syringes. Plasma separation and sample storage was performed as described above.

### AA analysis

Analysis of the AA concentration in plasma and urine was performed at the FGCZ (Functional Genomic Centre Zurich) using the Mass Track AA Analysis Application Solution and ACQUITY ultra performance liquid chromatography (UPLC; Waters, Milford, MA, USA) in accordance with the manufacturer's instructions. Plasma and urine samples were deproteinized by sulphosalicylic acid (10%) precipitation prior to UPLC.

### Statistical analysis

Data were plotted and analysed using Prism, versions 5.0 and 6.0 (GraphPad Software Inc., San Diego, CA, USA; RRID:SCR_002798). Differences between the mean values of groups were assessed with an unpaired Student's *t* test, as well as one‐way or two‐way ANOVA followed by a Bonferroni multiple comparison test, as indicated where appropriate. *P* < 0.05 was considered statistically significant. Data are shown as individual values with the mean ± SD. Exact numerical values for the mean ± SD and *P* values for each dataset are provided in the Supporting information (Statistical Summary Document).

## Results

### Global and intestinal LAT4 KO induced after the postnatal phase

**Figure 2:**
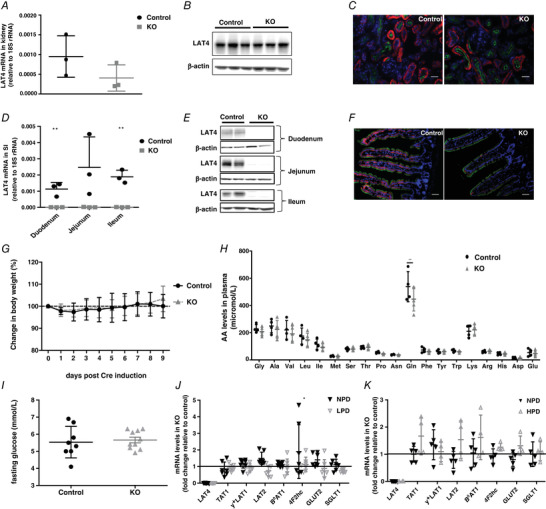
Characterization of the global LAT4 KO induced past the postnatal phase *A*–*C*, LAT4 KO in the kidney was almost 50% efficient at the mRNA level (*A*) but negligible at the protein level as observed by WB (*B*) and IF (*C*). Basolateral LAT4 is shown in red, luminal B^0^AT1 (SLC6A19) in green and nuclei in blue (DAPI). Scale bars = 50 μm. *D*–*F*, LAT4 KO efficiency in the SI was almost 98% efficient at both the mRNA (*D*) and protein levels (*E* and *F*). Labelling of colours for IF is as indicated in (*C*). Scale bar = 10 μm. *G*, changes in body weight expressed as a percentage. Fasting (*H*) AA concentrations and (*I*) glucose levels in plasma. *J* and *K*, relative gene expression of other known basolateral and luminal neutral AA and glucose transporters in KO mice subjected to TRF with (*J*) normal (NPD) or low (LPD) protein diet (in *LAT4^f/f^ Rosa26‐CreER^T2+^* mouse model) and (*K*) normal (NPD) or high protein diet (HPD) (in *LAT4^f/f^ Villin‐CreER^T2+^* mouse model). Values are normalized to those of control mice within each group. Data are shown as the mean ± SD (*n* = 3–10). *D*, unpaired Student's *t* test. *H*, one‐way ANOVA with a Bonferroni *post hoc* test. *J*, two‐way ANOVA with a Bonferroni *post hoc* test, ^*^ *P* < 0.05; ^**^ *P* < 0.01.

Constitutional LAT4 global KO in mice lead to a severe phenotype with early postnatal death (Guetg *et al*. 2015). To investigate whether LAT4 KO past the postnatal phase also leads to a severe phenotype, we generated an inducible global KO mouse model for LAT4 (*LAT4^f/f^ Rosa26‐CreER^T2+^*) by crossing *LAT4^f/f^* mice with *Rosa26‐CreER^T2+^* mice (Fig. 1). LAT4 KO was induced in these mice at 8 weeks of age by administering tamoxifen for five consecutive days. Ten days after the first tamoxifen injection, animals were killed and LAT4 KO efficiency tested in different organs, in particular in the SI and kidney (Fig. 2). In the kidney, the induced LAT4 KO was only ∼50% efficient at the mRNA level (Fig. 2 *A*) and negligible at the protein level (Fig. 2 *B* and *C*). By contrast, KO of LAT4 was ∼98% efficient in the SI at both mRNA (Fig. 2 *D*) and protein levels (Fig. 2 *E* and *F*). This LAT4 deletion induced in adult intestine remained stable even after 1 month post tamoxifen treatment (data not shown). Interestingly, this deletion of LAT4 in the SI induced past the postnatal phase did not lead to any change in body weight gain, fasting plasma AA or glucose levels (Fig. 2 *G–I*), nor did it lead to any visible phenotype.

To investigate the impact of LAT4 deletion in adult mouse SI in more detail, we additionally generated an intestine‐specific inducible LAT4 KO mouse model (*LAT4^f/f^ Villin‐CreER^T2+^*) which, at the level of the intestine, was found to be very similar to the global inducible model described above (∼98% LAT4 deletion at mRNA and protein levels; model further described below).

To challenge the intestinal AA absorption in adult mice lacking intestinal LAT4, we subjected animals to diets containing different amounts of proteins, namely low protein (8% = LPD), normal (18% = NPD) and high protein (40% = HPD) diets. Based on previous observations that the expression of various nutrient transporters are more responsive to food entrainment than just central circadian rhythm entrained by light, we subjected mice to TRF with access to food only for 8 h during the active phase (from ZT12 to ZT20) and killed them either at the start of their active phase (ZT12) or at the start of their inactive phase (ZT0) (Hatori *et al*. 2012; Longo & Panda 2016). Analyses of the expression of different basolateral (TAT1, y^+^LAT1, LAT2, 4F2hc, GLUT2) and apical (B^0^AT1 and SGLT1) membrane AA and glucose transporters revealed no significant compensatory changes at the mRNA level in the SI of LAT4 KOs at both ZT12 (Fig. 2 *J* and *K*) and ZT0 (data not shown). Taken together, our data clearly suggest that the KO of LAT4 in SI induced past the postnatal phase did not lead to any obvious phenotype, nor to a significant compensation at the level of the mRNA of other studied nutrient transporters.

### Constitutional intestine‐specific LAT4 KO

**Figure 3:**
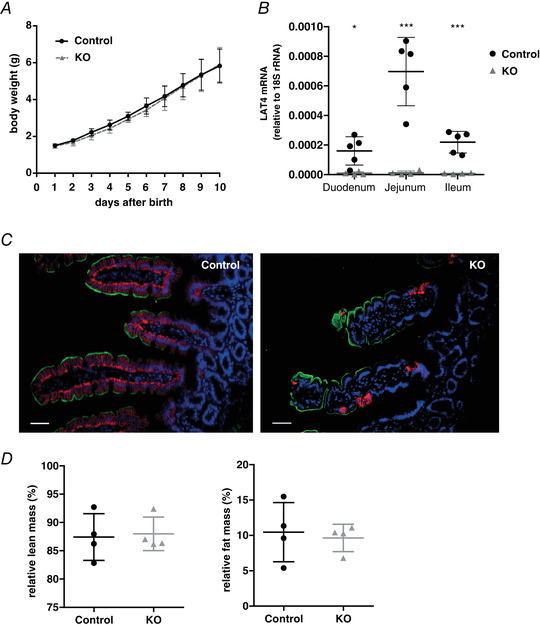
Characterization of constitutional intestine‐specific LAT4 KO mice *A*, growth curve of KO (*LAT4^f/f^ Villin‐Cre^+^*) and control mice (*LAT4^f/f^ Villin‐Cre^−^*). *B*, real‐time reverse transcriptase‐PCR quantitation shows an 85–95% decrease of LAT4 mRNA in the SI. *C*, LAT4 protein expression by IF imaging also shows an 85–95% decrease in the SI of LAT4 KO mice compared to control mice. Basolateral LAT4 shown in red, luminal B^0^AT1 (SLC6A19) in green and nuclei in blue (DAPI staining). *D*, changes in body composition expressed as a percentage of total body mass. Data are shown as the mean ± SD (*n* = 4). *B*, unpaired Student's *t* test, ^*^ *P* < 0.05; ^***^ *P* < 0.001. Scale bars = 50 μm.

Although the KO of LAT4 in the SI past the postnatal phase did not affect the general well‐being of mice, we could not completely nullify the hypothesis that LAT4 KO in the SI could have led to the lethal phenotype of LAT4 global KO pups, unless we confirm the role of LAT4 in the SI during pre‐ and postnatal development. Accordingly, we generated constitutional intestine‐specific LAT4 KO (*LAT4^f/f^ Villin‐Cre^+^*) mice by crossing the *LAT4^f/f^* mice with mice constitutively expressing Cre recombinase in the intestine: *Villin‐Cre^+^* (El Marjou *et al*. 2004) (Fig. 1). KO and control littermate pups obtained by crossing *LAT4^f/wt^ Villin‐Cre^+^* mice with *LAT4^f/f^* mice were monitored from birth. Surprisingly, no change in body weight (Fig. 3 *A*), body composition (Fig. 3 *D*) or general behaviour was detected between the genotypes even up to 2 months after birth. Mice were then killed and the efficiency of LAT4 KO was studied. LAT4 KO was found to be 85–95% efficient in the SI at both mRNA and protein levels (Fig. 3 *B* and *C*). LAT4 KO efficiency was also validated in 5‐day‐old pups and gave identical results (data not shown).

Using 8‐week‐old mice, we further analysed the impact of LAT4 deletion on AA transport along the intestine by gavaging them with an AA mix containing radioactively labelled ^3^H‐Leu (a LAT4 substrate) and ^14^C‐Lys (not a LAT4 substrate) as tracers. Blood was collected from the tail vein at six different time points: 10 min before, 2, 5, 10, 20 and 60 min after gavage, after which mice were killed and the distribution of radiolabelled AAs in the luminal content of the intestine, in the intestinal tissue and in the blood plasma was analysed.

LAT4 KO mice displayed a strong accumulation of the LAT4 substrate Leu but not of Lys in the initial segment (SI1) of the small intestinal tissue harvested 60 min after the gavage (Fig. 4 *A*, *B* and *N*). In addition, LAT4 KOs displayed higher levels of both Leu and Lys remaining in the luminal content of the third (late jejunum) segment SI3, whereas, in wild‐type mice, they were higher in the last segment SI4, denoting a delay in the overall bolus movement in the KOs (Fig. 4 *C* and *D*). Killing the mice at 20 minutes after the oral gavage also revealed a similar absorption defect of ^3^H‐Leu in SI1 (Fig. 4 *E* and *N*) with a delayed bolus movement, which, in this case, was still in segments SI1 and SI2 instead of SI3 and SI4, respectively (Fig. 4 *G*). When we gavaged mice with radiolabelled ^14^C‐Met (another LAT4 substrate), we observed results similar to those obtained with ^3^H‐Leu, further confirming the observations made with ^3^H‐Leu (Fig. 4 *F* and *H*).

Figure 4Functional analysis of AA transport in the SI of constitutional intestine‐specific LAT4 KO mice
*A*–*D*, distribution of radiolabelled AAs [^3^H‐Leu (LAT4 substrate) and ^14^C‐Lys (not a LAT4 substrate)] in tissue and luminal content of the SI of LAT4 KO (grey triangles) (*LAT4^f/f^ Villin‐Cre^+^*) and control (black dots) (*LAT4^f/f^ Villin‐Cre^−^*) mice 60 min after they were given by gavage as tracers in an AA mix. *E*–*H*, distribution of radiolabelled AAs [^3^H‐Leu and ^14^C‐Met (both LAT4 substrates)] as for (*A*) to (*D*), but measured 20 min after gavage. The amount of gavaged AAs found in intestinal tissue (*A*, *B*, *E* and *F*) is indicated relative to the tissue weight and AAs measured in the luminal content as nmol per segment of the small intestine (SI). *I*–*K*, time course analysis of the appearance in blood plasma of radiolabelled AAs (^3^H‐Leu, ^14^C‐Lys and ^14^C‐Met) and (*L* and *M*) of paracellularly transported molecules (^3^H‐mannitol and fluorescence labelled FITC‐dextran) given by oral gavage. Values are given as pmol μL^–1^ plasma (*I*–*L*) or as arbitrary fluorescence units (*M*). *N*, schematic representation of intestinal absorption defect and delayed bolus movement in LAT4 KO mice. Data are shown as the mean ± SD (*n* = 5–6). Two‐way ANOVA with a Bonferroni *post hoc* test, ^*^ *P* < 0.05; ^**^ *P* < 0.01; ^***^ *P* < 0.001.

**Figure:**
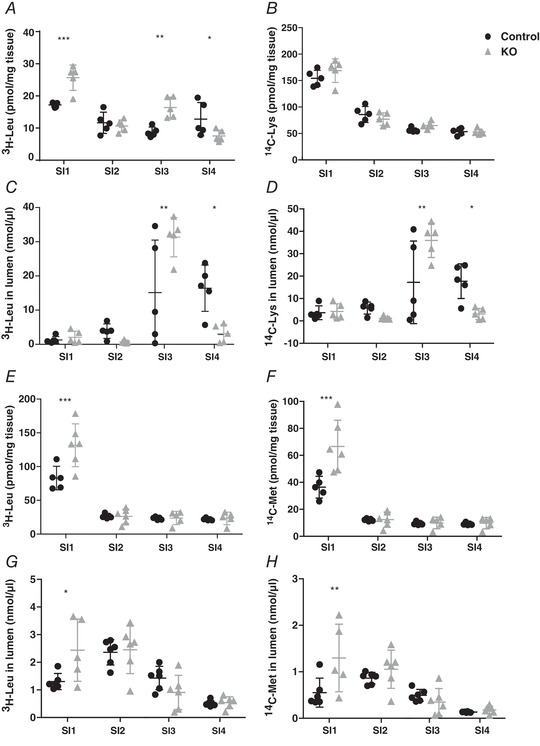

**Figure:**
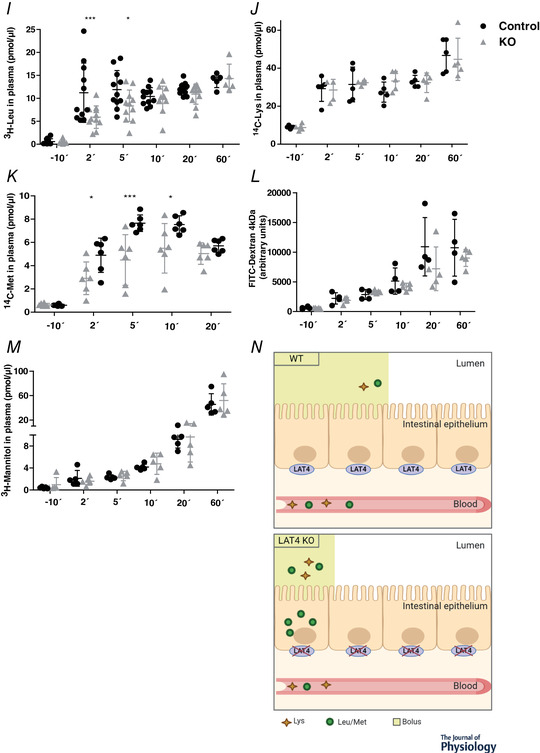

Time course measurements of the appearance of labelled AAs in the blood after gavage showed that LAT4 substrates Leu and Met but not Lys (which is not a LAT4 substrate) appeared with a small delay in the blood of LAT4 KOs compared to control littermates (Fig. 4 *I–K*). At later time points, Leu (after 10 min) and Met (after 30 min) were abundant in the blood plasma of LAT4 KOs to a similar extent as in the controls, indicating that they were absorbed anyway, suggesting a possible compensatory (or redundant) role of other basolateral transporters and/or of a paracellular leak. Assessing a possible paracellular leak in the intestine of these mice using the relatively high molecular weight marker FITC‐dextran (4 kDa) and smaller molecules such as ^3^H‐Mannitol (182 Da) did not reveal a significant difference between the KO and the control group, although it demonstrated that such a leak is present and suggested a general increase in intestinal paracellular permeability with time, irrespective of genotype (Fig. 4 *L* and *M*).

### TAT1‐LAT4 dKO in SI

**Figure 5:**
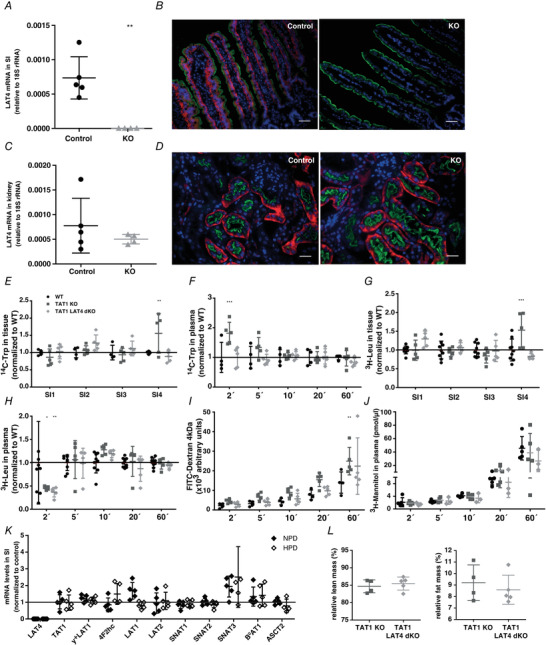
dKO of the basolateral uniporters TAT1 and LAT4 in the SI LAT4 KO efficiency in the (*A* and *B*) small intestine and (*C* and *D*) kidney of *LAT4^f/f^ Villin‐CreER^T2+^* mice investigated by quantitative PCR and IF. Basolateral LAT4 shown in red, luminal B^0^AT1 (SLC6A19) in green and nuclei in blue (DAPI staining). Scale bars (*B*) = 50 μm and (*D*) = 25 μm. *E*–*H*, amount of radiolabelled Trp and Leu in intestinal tissue (*E* and *G*) 60 min after the oral gavage of an AA mix containing the radiolabelled tracer AAs and time course of their appearance in blood plasma (*F* and *H*). Values of TAT1‐LAT4 dKO (*TAT1^−/−^ LAT4^f/f^ Villin‐CreER^T2+^*) and TAT1 KO (*TAT1^−/−^*) are normalized to that of the WT controls. *I* and *J*, assessment of paracellular leak using fluorescence labelled FITC‐dextran (*I*) or radiolabelled mannitol (*J*). *K*, relative gene expression of other known basolateral and luminal neutral AA transporters in dKO mice (values normalized to that of single TAT1 KO mice in each group) subjected to a normal (NPD) or high protein diet (HPD). *L*, changes in body composition expressed as percentage of total body mass. Data are shown as the mean ± SD (*n* = 4–5). *A*, unpaired Student's *t* test. *E*–*I*, two‐way ANOVA with a Bonferroni *post hoc* test, ^*^ *P* < 0.05; ^**^ *P* < 0.01; ^***^ *P* < 0.001.

We hypothesized that deletion of TAT1 (the only other known basolateral uniporter in SI), in addition to that of LAT4, would potentially interrupt the directional transport of AAs through the basolateral membrane and would eliminate the potential compensatory/redundant role of TAT1. Hence, we generated an intestine‐specific dKO for LAT4 and TAT1, *TAT1^−/−^ LAT4^f/f^ Villin‐CreER^T2+^* (further referred as TAT1‐LAT4 dKO), by crossing the global constitutional TAT1 null mice (*TAT1^−/−^*) with inducible intestine‐specific LAT4 KO mice (*LAT4^f/f^ Villin‐CreER^T2+^*). LAT4 KO in this model was induced by giving i.p. injections of tamoxifen for 5 consecutive days and allowing for a minimum recovery period of 10 days after the first injection. The induced LAT4 KO was almost 100% efficient in the SI and negligible in the kidney at both mRNA and protein levels (Fig. 5 *A–D*). The KO efficiency of TAT1 in the TAT1^−/−^ mice has been described previously (Mariotta *et al*. 2012). Interestingly, induction of LAT4 KO in this model did not lead to any significant changes in the body weight gain (data not shown) or body composition (Fig. 5 *L*) of these mice compared to their control littermates.

To precisely investigate the functional impact of double deletion of LAT4 and TAT1 on SI AA absorption, we used a gavage protocol similar to that described above for constitutional intestine‐specific LAT4 KO mice (*LAT4^f/f^ Villin‐Cre^+^*). We gavaged an AA mix containing radiolabelled ^14^C‐Trp (a TAT1 substrate) and ^3^H‐Leu (a LAT4 substrate) to TAT1‐LAT4 dKO, TAT1 KO and wild‐type like LAT4^f/f^ (WT) mice. In the case of TAT1 substrate, ^14^C‐Trp, we observed its significant accumulation in the distal small intestinal tissue (SI4) in TAT1 KO mice, although there were no significant changes in the TAT1‐LAT4 dKO mice, which showed only a mild trend towards Trp accumulation in SI2 (Fig. 5 *E*). In addition, TAT1 KO mice displayed a stronger early appearance of gavaged Trp in the plasma, which was absent in the TAT1‐LAT4 dKOs that closely resembled WT controls (Fig. 5 *F*). In the case of LAT4 substrate Leu, TAT1 KO but not TAT1‐LAT4 dKO mice displayed a strong accumulation in the small intestinal tissue of SI4, although Leu is not a substrate of the aromatic AA transporter TAT1 (Fig. 5 *G*). The appearance, and thus the absorption, of Leu into blood was also shortly delayed in TAT1 KOs (only at the initial time point of 2 min after gavage), and this effect was also present in the TAT1‐LAT4 dKOs (Fig. 5 *H*). At later time points, the dKOs behaved very similarly to the WT controls. Taken together with the results obtained from intestine‐specific LAT4 KO mice described above, single deletions of LAT4 or TAT1 led to stronger defects in Leu absorption than the double deletion of TAT1 and LAT4, suggesting a possible compensatory mechanism via another AA transporter or a paracellular leak that is turned on specifically in the absence of both uniporters. However, probing for a compensatory increase in paracellular leak (using paracellularly transported molecules such as mannitol and FITC‐dextran) (Fig. 5 *I*) or investigating the gene expression of other known AA transporters (Fig. 5 *J*) revealed no significant difference between the genotypes.

### Kidney tubule‐specific LAT4 KO

**Figure 6:**
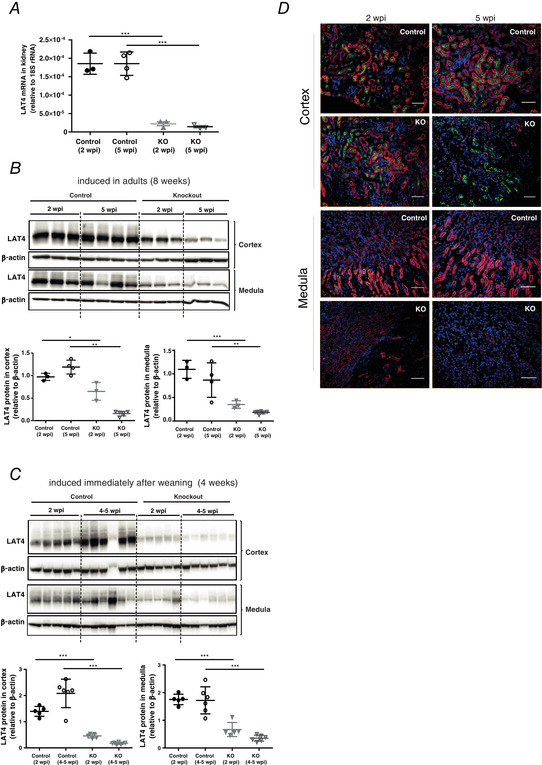
Validation of LAT4 KO efficiency in inducible kidney tubule‐specific LAT4 KO mice *A*, real‐time reverse transcriptase‐PCR quantitation of LAT4 mRNA and (*B*) western blotting analysis of LAT4 protein expression and its respective quantification in control (*LAT4^f/f^ Pax8rtTA^−^LC1^+^*) and KO (*LAT4^f/f^ Pax8rtTA^+^ LC1^+^*) mice at 2 and 5 weeks post induction (wpi) of LAT4 KO when induced at 8 weeks of age. *C*, LAT4 protein expression investigated by WB at 2 and 4 wpi of LAT4 KO when induced at 4 weeks of age. Induced LAT4 KO was almost 90% efficient already at 2 wpi at the mRNA level but reached 90% efficiency at the protein level only after 4–5 wpi. *D*, LAT4 protein expression in the kidney cortex and medulla analysed by immunofluorescence at 2 and 5 wpi of LAT4 KO when induced at 8 weeks of age (inducing LAT4 KO at 4 weeks of age gave similar results). LAT4 KO at the protein level is very efficient in the kidney cortex (PTs) and medulla only after 4–5 wpi of LAT4 KO. Basolateral LAT4 shown in red, luminal B^0^AT1 (SLC6A19) (PT marker) in green and nuclei in blue (DAPI staining). Scale bars = 25 μm. Data are shown as the mean ± SD (*n* = 3–6). *A*–*C*, one‐way ANOVA with a Bonferroni *post hoc* test, ^*^ *P* < 0.05; ^**^ *P* < 0.01; ^***^ *P* < 0.001.

To investigate the specific role of LAT4 in the kidney tubular reabsorption of AAs, we crossed our *LAT4^f/f^* mice with an inducible kidney tubule‐specific Cre mouse: *Pax8‐rtTA^+^/LC‐1^+^* (Traykova‐Brauch *et al*. 2008) as shown in Fig. 1. LAT4 KO in the kidney tubules of this model (*LAT4^f/f^ Pax8‐rtTA^+^ LC1^+^*) was induced by administering 2 mg mL^–1^ doxycycline in 2% sucrose via drinking water for 10 days to 8‐ or 4‐week‐old mice. To optimize the LAT4 KO induction procedure and test for its efficiency, we killed one set of mice at 2 weeks post induction (wpi). The second set of mice received an additional short doxycycline treatment from day 28 to 30 and was killed at 5 wpi. Inducing LAT4 KO in 8‐week‐old mice resulted in almost 90% efficient LAT4 mRNA deletion already at 2 wpi (Fig. 6 *A*). However, at 2 wpi, KO of LAT4 protein was only ∼30% and 60% efficient in the cortex and medulla, respectively. At 5 wpi, LAT4 protein deletion reached almost 90% and 80% in cortex and medulla, respectively (Fig. 6 *B*). Attempts to improve LAT4 KO efficiency by inducing it immediately after weaning did not lead to a marked increase in the KO efficiency at the protein level at 2 wpi, but only at 4–5 wpi, suggesting that LAT4 protein is indeed very stable in the kidney, especially in the cortex (Fig. 6 *C*). IF on kidney sections for LAT4 and B^0^AT1 (a PT‐specific luminal membrane AA transporter), also showed an efficient KO of LAT4, especially in the PTs only after 4–5 wpi (Fig. 6 *D*).

**Figure 7:**
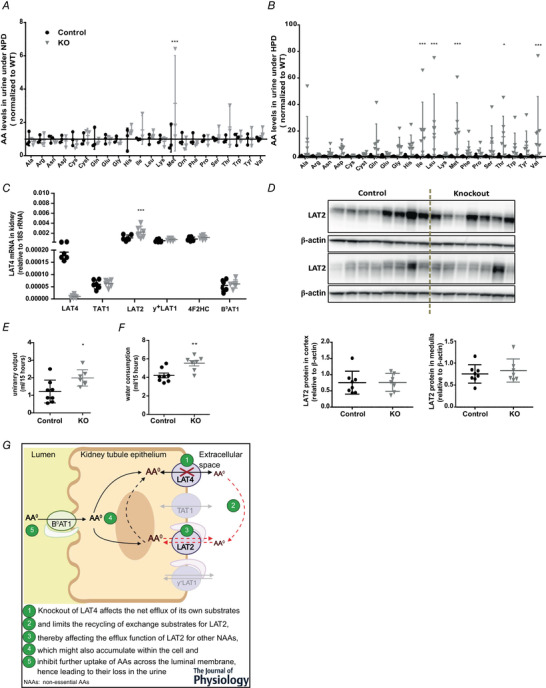
Functional analysis of AA reabsorption in mice with inducible kidney tubule‐specific LAT4 KO *A* and *B*, AA concentrations in the urine of KOs (LAT4^f/f^ Pax8rtTA^+^ LC1^+^) compared to control mice (*LAT4^f/f^ Pax8rtTA^−^ LC1^+^*) subjected to (*A*) normal (NPD) or (*B*) high protein diet (HPD) at 4–5 weeks post induction of LAT4 KO. *C*, relative gene expression of other known basolateral and luminal neutral AA transporters in the kidney reveals up‐regulation of the basolateral antiporter LAT2. *D*, WB analysis of LAT2 protein in the kidney cortex and medulla and its quantification relative to β‐actin. *E*, urinary volume and (*F*) water consumption rate in LAT4 KO and control mice fed HPD. *G*, schematic representation of the reabsorption defect in kidney of LAT4 KOs that affects transport of LAT4, as well as LAT2 substrates. Data are shown as the mean ± SD (*n* = 3–8). *A*–*C*, two‐way ANOVA with a Bonferroni *post hoc* test. *E* and *F*, unpaired Student's *t* test, ^*^ *P* < 0.05; ^**^ *P* < 0.01; ^***^ *P* < 0.001. [Color figure can be viewed at wileyonlinelibrary.com]

Given the highly efficient KO of LAT4 protein in kidney PTs, the region where most AAs are known to be reabsorbed, we tested for a possible defect in tubular AA reabsorption by collecting spot urine and plasma samples from mice during the absorptive phase at ZT14–16. Mice were subjected to TRF for 3 days before the sample collection to ensure that they start feeding only at ZT12. As shown in Fig. 7 *A*, analysing AA concentrations in the urine of these mice revealed an almost three‐fold increase in the urinary excretion of the LAT4 substrate Met (and a trend toward increase of other LAT4 substrates Leu, Ile and Val) under NPD (LAT4 KO induced at 8 weeks of age). However, subjecting mice to a HPD (LAT4 KO induced at 4 weeks of age) to challenge LAT4's maximal transport capacity, led to a more obvious phenotype with marked increase in the urinary excretion of not only LAT4 substrates (Met, Leu, Ile, Val, increased by almost 20‐fold, and Phe increased by five‐fold), but also LAT2 substrates (Ala, Asn, Gln, Gly, His, Ser, Thr, Trp and Tyr increased by three‐ to 15‐fold) and, additionally, of Pro and Asp (by three‐ to seven‐fold) (Fig. 7 *B*). We also observed a corresponding increase in LAT2 mRNA levels in LAT4 KO mice (Fig. 7 *C*), although we did not detect a concordant increase in LAT2 protein (Fig. 7 *D*). By contrast to urinary AA levels, we observed no significant differences in plasma AA concentrations between the KOs and controls both under NPD and HPD conditions (data not shown). Performing similar studies and maintaining mice in metabolic cages overnight to collect urine and plasma samples led to similar results. In accordance to the increased aminoaciduria, urinary volume and water consumption rate was also increased in LAT4 KO mice (Fig. 7 *E* and *F*). Overall, our observations clearly demonstrate an important role for LAT4 in the tubular reabsorption of AAs in the kidney (Fig. 7 *G*).

### Partial LAT4 deletion in TAT1 KO

**Figure 8:**
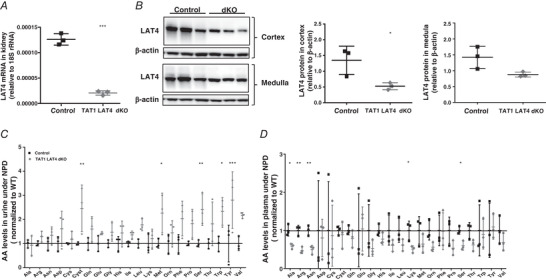
Characterization of inducible global TAT1‐LAT4 dKO mice *A*, real‐time reverse transcriptase‐PCR quantitation shows an almost 85% decrease of LAT4 mRNA (*B*) WB analysis of LAT4 protein expression and its respective quantification relative to β‐actin shows an almost 60% reduction in LAT4 protein in the kidney of TAT1‐LAT4 dKO (*TAT1^−/−^ LAT4^f/f^ Rosa26‐CreER^T2+^*) compared to control: TAT1 KO (*TAT1^−/−^ LAT4^f/f^ Rosa26‐CreER^T2−^*) mice. AA concentrations in the (*C*) urine and (*D*) plasma of inducible global TAT1‐LAT4 dKO mice subjected to a normal protein diet (NPD). Values normalized to that of single TAT1 KO mice. Data are shown as the mean ± SD (*n* = 3). *A*, *B* and *D*, unpaired Student's *t* test. *C*, two‐way ANOVA with a Bonferroni *post hoc* test; ^*^ *P* < 0.05; ^**^ *P* < 0.01; ^***^ *P* < 0.001.

To investigate whether simultaneous deletion of LAT4 and TAT1 in the kidney would act synergistically on aminoaciduria or lead (as observed in the SI) to no major effect on AA transport (Fig. 5), we generated an inducible *Rosa26‐CreER^T2^*‐mediated global LAT4 KO in the constitutional global TAT1 KO mouse, *TAT1^−/−^ LAT4^f/f^ Rosa26‐CreER^T2+^* (further referred as TAT1‐LAT4 global dKO) (Fig. 1). Based on our observations that the half‐life of LAT4 protein is relatively long in the kidney, LAT4 KO was induced immediately after weaning by giving i.p. injections of tamoxifen 2 mg/25 g body weight for 5 consecutive days, followed 10 days later by a second tamoxifen dose of 3 mg/25 g body weight for 3 consecutive days. Experiments were performed another 10 days later (1 month after first tamoxifen injection). With this procedure, LAT4 KO efficiency in the kidney reached almost 85% at the mRNA level (Fig. 8 *A*) and ∼60% at the protein level (Fig. 8 *B*).

Kidney tubular AA reabsorption was tested in this model by measuring AAs in urine samples collected over a 16 h period (from ZT12 to ZT4) from mice subjected to NPD. Kidney AA reabsorption was more strongly affected in these partial global dKO mice as revealed by a 1.5‐ to three‐fold increase in the urinary excretion of almost all AAs (except Ala, Arg, Asn, Cys and Lys) compared to the TAT1 KO littermates (Fig. 8 *C*), despite the fact that the plasma concentration of most of the AAs (except Asp, Cys, Cyst, Gln and Glu and the aromatic AAs) was reduced by around one‐half (Fig. 8 *D*). These observations show that a partial global deletion of LAT4 [60% reduction in the kidney (Fig. 8 *B* and *C*) and ∼98% in the intestine (Fig. 2 *D–F*)], together with complete global TAT1 deletion, leads to profound changes in body AA homeostasis even under NPD and, specifically, to a synergistic decrease of renal AA reabsorption.

## Discussion

### Lack of intestinal LAT4 is not lethal but affects AA absorption and gastrointestinal motility in mice

To investigate the role of LAT4 past the postnatal phase, we used an inducible global LAT4 KO mouse model (*LAT4^f/f^ Rosa26‐CreER^T2+^*). Induced LAT4 KO was very efficient and stable in the SI at the mRNA and protein levels (almost 100%) (Fig. 2). This indicates an efficient targeting of the stem cells located at the bottom of the crypts because the intestinal epithelium in mice is rapidly renewed every 4–7 days (van der Flier & Clevers 2009). By contrast, LAT4 deletion in kidneys (Fig. 2 *A–C*) and other organs such as the brain, adrenal glands and adipose tissue (data not shown) was poor.

The efficient LAT4 KO in SI, however, did not affect the growth or general behaviour of mice. A more detailed investigation of the SI function in *LAT4^f/f^ Rosa26‐CreER^T2+^* mice suggested an AA absorption defect because the LAT4 substrate Leu was found to be accumulated within the initial segments of the SI (duodenum) (data not shown), similar to the observation made previously by Guetg *et al*. (2015) in LAT4 global KO pups. However, unlike in the LAT4 global KO pups, the plasma AA levels of induced global LAT4 KO mice were almost unchanged, except for a relatively small decrease in l‐glutamine. This altered l‐glutamine level may denote altered metabolism and/or basolateral transport in enterocytes because the intestine is a major consumer of l‐glutamine (Hartmann & Plauth 1989) and glutamine transporter LAT2 presumably co‐operates with LAT4 for its transport (Ramadan *et al*. 2007).

Several recent studies have reported the expression of LAT4 in rat and mouse pancreatic islet alpha and beta cells (Cheng *et al*. 2016; Kim *et al*. 2017). The lack of plasma glucose level alteration in our inducible global LAT4 KO mice probably suggests an inefficient KO of LAT4 in these cells. It can thus not be excluded that LAT4 plays an important role for the regulation of glucagon and/or insulin secretion and that its lack in pancreatic islet cells may have played an important role for the metabolic status and lethality of global KO pups.

We next investigated whether other known intestinal basolateral neutral AATs could compensate for the lack of LAT4 in the SI. Even under conditions of time‐restricted feeding (to synchronize the expression of nutrient‐responsive genes) (Hatori *et al*. 2012; Longo & Panda 2016) and under different protein diets (low 8%, and normal 18%), we did not observe any compensatory change at the mRNA level of the tested AA transporters. We also observed no compensatory changes under a HPD (40% protein), using an intestine‐specific inducible LAT4 KO mouse model (*LAT4^f/f^ Villin‐CreER^T2+^*), which was also almost 100% efficient in knocking out LAT4 in the SI without affecting the kidney, unlike other Villin promoter constructs that are also active in the kidney (Pinto *et al*. 1999). We can nevertheless not exclude a compensatory change at the level of other transporters or even at the level of the tested ones, if occurring by post‐transcriptional modifications, such as LAT4 that itself was shown to undergo post‐translational regulation in the context of food anticipation, specifically by serine (de)phosphorylation (Oparija *et al*. 2019).

Taken together, our attempt to induce an efficient global deletion of LAT4 past the postnatal phase was not successful because the induced LAT4 deletion was not uniformly efficient in target organs, preventing us from understanding whether the generalized lack of this AA transporter would lead to a severe malnutrition‐like phenotype also in later life, as it did in newborn mice (Guetg *et al*. 2015). However, our results clearly demonstrate that LAT4 deletion in the SI past the postnatal phase leads only to a mild absorption defect without affecting the general well‐being of mice and without inducing a compensatory increase of other known basolateral AATs at the mRNA level.

Further analysis of the role of LAT4 in intestine during early development (embryonic and postnatal phase) using a constitutional intestine‐specific LAT4 KO mouse model (*LAT4^f/f^ Villin‐Cre^+^*) revealed no changes in body weight gain, body composition or general behaviour, despite a highly efficient KO of LAT4 in the SI. As a result of the length and larger surface area of the SI, it was technically difficult to measure the levels of non‐absorbed amino acids (which is comparatively easier to measure in the kidney with the low background of AAs excreted in the urine). Therefore, we followed the fate of gavaged radioactive amino acids in the intestinal content, the mucosal cells and the blood. Constitutional intestine‐specific LAT4 KO mice displayed a strong accumulation of Leu and Met (LAT4 substrates) but not Lys (not LAT4 substrate) within the cells of the initial segments of the SI (Fig. 4), similar to that observed by Guetg *et al*. (2015) in the LAT4 global KO mice, suggesting an active apical uptake of these AAs but a defective basolateral efflux (Fig. 4 *N*). This accumulation also led to the retention of LAT4 substrates within the lumen of the intestine that presumably caused the delay of intestinal bolus movement observed in the KOs (both when measuring at 20 and 60 min after the oral gavage of the AAs) (Jordi *et al*. 2014). Taken together, these changes could have caused the delayed absorption of the LAT4 substrates Leu and Met into the blood (decreased only during the initial time points: 2 and 5 min after the oral gavage), although they did not affect the absorption of the cationic AA Lys. This early delay in absorption of substrate AAs is apparently opposed to the slower effect observed in previous studies on mice lacking the luminal amino acid transporter B^0^AT1 (Javed and Broer, 2018). This difference might be explained by the fact that we measured the appearance of gavaged radiolabelled AA tracers in the plasma and not the postprandial accumulation of AAs. The discrete time differences between the Leu and Met absorption peaks may be related to the difference in affinity of LAT4 for these AAs (Bodoy *et al*. 2005). Overall, these changes in early absorption and gastrointestinal motility observed upon intestinal LAT4 KO did not prevent LAT4 substrates from finally reaching the blood. This remaining absorption did not appear to be mediated by an increased expression of other known basolateral AATs (as observed in inducible intestine‐specific LAT4 KOs), nor by an increase of the paracellular permeability, because we did not observe an increase in absorption of the paracellular markers FITC‐dextran (4 kDa) (Gupta & Nebreda 2016; Woting & Blaut 2018) and mannitol (Ménard *et al*.2010; Mnard *et al*. 2012). Overall, our observations suggest that LAT4 in intestine plays a role in absorption and regulating gut motility (Fig. 4 *N*) but does not have an impact on growth or survival of mice.

### LAT4 TAT1 dKO in the intestine suggests the presence of another directional AA efflux pathway in the basolateral membranes of SI epithelia

To eliminate a potential compensation for the lack of intestinal LAT4 by TAT1, the only other known basolateral AA uniporter in SI, we generated an inducible intestine‐specific LAT4 KO in TAT1 global KO background (TAT1 LAT4 dKO) (Fig. 5). Given the parallel role of the two basolateral uniporters LAT4 and TAT1, we had anticipated that their double deletion in the SI would lead to a massive absorption defect, although we did not observe any significant alteration in the absorption of Leu (LAT4 substrate) or Trp (TAT1 substrate) in the TAT1 LAT4 dKOs compared to WTs. This was particularly surprising, because the same *in vivo* absorption experiments performed in TAT1 or LAT4 single KO mice (Fig. 4) had revealed clear alterations in the distribution of radiolabelled AAs among the intestinal lumen, tissue and blood plasma. Higher levels of Trp in the plasma of TAT1 KOs (as opposed to lower levels of substrates during an absorption defect) are possibly related to the fact that absence of this transporter in the liver interferes with the clearance of aromatic AAs during their first passage through the liver (Mariotta *et al*. 2012). These observations suggest the presence of another unidentified directional AA efflux pathway in the basolateral membrane of SI enterocytes that is activated only in the absence of both LAT4 and TAT1. Assessing the RNA expression of known basolateral AA transporters or testing for a potential paracellular leak functioning as a compensatory mechanism did not show any major differences between dKO and WT mice. Thus, we hypothesize that such an AA efflux pathway could be a yet unidentified transporter (uniporter) or a non‐selective, possibly volume‐regulated channel. Yet another possibility is that some of the AA antiporters expressed in SI enterocytes (e.g. LAT2, y^+^LAT1 or ASCT2) would, in dKO conditions, switch to an alternative kinetic mode of non‐obligatory exchange and function as efflux pathway (Fuchs & Bode 2005). Some of these antiporters considered to function as obligatory exchangers (based on measurements made at 20°C), may contribute to a small leak (uniport mode transport) at 37°C (Pineda *et al*. 1999), which could explain at least part of the directional transport observed in TAT1 LAT4 dKO.

### LAT4 KO in kidney leads to a large neutral aminoaciduria

As shown in previous studies, LAT4 is quite highly expressed in different kidney tubule segments (Bodoy *et al*. 2005; Guetg *et al*. 2015), although its role in these tubules was not known. As the kidney plays a key role in maintaining AA homeostasis by reabsorbing most AAs, preventing their loss in the urine, it could not be excluded that LAT4 in the kidney contributed significantly to the lethal phenotype of the LAT4 global KO pups. Since our inducible global LAT4 KO mouse model (*LAT4^f/f^ Rosa26‐CreER^T2+^*) turned out to be inefficient in knocking out LAT4 in the kidney, we generated an inducible kidney tubule‐specific LAT4 KO mouse model (*LAT4^f/f^ Pax8‐rtTA^+^ LC1^+^*) using a system known to have a higher penetrance and specificity for the entire kidney tubular epithelium (Fig. 6) (Traykova‐Brauch *et al*. 2008). With this model, we observed that the half‐life of LAT4 protein in the kidney is relatively long, especially in the cortex, the region enriched for PTs, which is also the site where almost all reabsorption of AAs from primary urine occurs (Makrides *et al*. 2014). Our observations also show that an efficient KO of LAT4 in kidney PTs leads to a significant loss of LAT4 substrate Met in the urine (and a trend toward loss of other LAT4 substrates), and a more spectacular reabsorption defect under HPD with a marked increase in the urinary excretion of not only LAT4 substrates, but also of LAT2 substrates (Fig. 7). These results clearly suggest functional co‐operation (dependency) of the antiporter LAT2 on LAT4 to mediate the transport of non‐essential AAs. Such co‐operation has yet only been demonstrated *in vitro* (Ramadan *et al*. 2007) and our findings represent the first evidence of such co‐operation *in vivo*. This co‐operation of LAT4 with LAT2 could also explain the altered levels of non‐essential AAs observed previously in the plasma and amniotic fluid of LAT4 global KO pups (Guetg *et al*. 2015). However, we did not detect any significant changes in the plasma AA levels in these mice (*LAT4^f/f^ Pax8‐rtTA^+^ LC1^+^*) suggesting that LAT4 KO in the kidney is not a major contributor to the overall altered AA homeostasis in LAT4 global KO pups. Taken together, our observations demonstrate that LAT4 plays a prominent role in the kidney PTs for the reabsorption of neutral AAs from the primary urine. Understanding the role of LAT4 in other kidney tubules such as the distal renal tubules, connecting tubules and collecting ducts still needs further investigation.

### Partial LAT4 KO in TAT1 global KO mice suggests an indispensable role for the uniporters LAT4 and TAT1 in tubular reabsorption of AAs

In an attempt to confirm whether TAT1 and LAT4 are the sole or major uniporters in the basolateral membranes of kidney PTs, we generated an inducible global LAT4 KO in TAT1 global KO background, *TAT1^−/−^ LAT4^f/f^ Rosa26‐CreER^T2+^* mouse model (TAT1 LAT4 global dKO). Because the half‐life of LAT4 protein is relatively high in the kidney, our attempts to induce its KO immediately after weaning and for longer time periods led to a significant reduction of LAT4 expression. Our observations show that knocking out both the uniporters in the kidney leads to a substantial aminoaciduria even under NPD, which included not only substrates of LAT4 and TAT1, but also other neutral AAs that are LAT2 substrates. This aminoaciduria, although quantitatively not impressive (1.5‐ to three‐fold for 11 AAs) is clearly more pronounced and broader than that observed for the global TAT1 single KO (Mariotta *et al*. 2012) or the much more complete (∼90%) kidney tubule‐specific LAT4 single KO (Pax8‐rtTA LC1‐driven) (Fig. 7). This synergistic effect strongly suggests that together TAT1 and LAT4 are indispensable for driving directional transport via the co‐expressed antiporters, in particular LAT2 in the kidney.

In addition, these TAT1 LAT4 global dKO mice also displayed about a two‐fold reduction in the level of many plasma AAs, including most neutral and cationic ones, compared to the TAT1 KO mice that were used as controls in this experiment, which is an effect that is not observed in the partial LAT4 KO (*LAT4^f/f^ Rosa26‐CreER^T2+^*) (Fig. 2) or inducible kidney‐specific LAT4 KO (*LAT4^f/f^ Pax8‐rtTA^+^ LC1^+^*) mice described above. We hypothesize that this reduction of plasma AA levels observed under NPD was largely caused by the synergistic effect of the lack of TAT1 and LAT4 in other tissues and not only by the amino acid loss as a result of the relatively mild intestinal and kidney (re)absorption defects.

Collectively, our results demonstrate that LAT4 is necessary for an optimal absorption of its substrate AAs in the SI, where it also plays a role in altering gastrointestinal transit (Table 4). In the kidney, LAT4 is shown to support the reabsorption of almost all neutral AAs (except cysteine) from the primary urine. Its partial KO in TAT1 null mice suggests that, together, these two basolateral uniporters are indispensable for amino acid reabsorption. However, the deletion of LAT4 in SI or kidney did not alter the body weight or body composition, nor did it lead to a severe phenotype unlike that observed previously in LAT4 global KO pups. Hence, this lethal malnutrition‐like phenotype could have been a result of the combined lack of LAT4 in SI and kidney together with its defect in other organs and cell types, such as the endocrine pancreas, brain and leukocytes, and/or via indirect effects, in particular at the level of metabolically active organs such as the liver.

**Table 4:** Summary of the different mouse models used and the corresponding observations

KO mouse models	KO efficiency	Question	Phenotype	Conclusion	Figure
Inducible global LAT4 KO (*LAT4^f/f^ Rosa26‐CreER^T2+^*)	In SI, almost 100% Low efficiency in kidney and other organs	Is LAT4 KO lethal past the postnatal phase?	Mild absorption defect in SI No changes in growth, plasma AA or glucose levels, no compensation by known AATs at RNA level	LAT4 KO in SI past postnatal phase is not lethal	Fig. 2
Inducible intestine‐specific LAT4 KO (*LAT4^f/f^ Villin‐CreER^T2+^*)	Almost 100%				Fig. 5
Constitutional intestine specific LAT4 KO (*LAT4^f/f^ Villin‐Cre^+^*)	Almost 85–95%	Role of intestinal LAT4 during pre‐ and postnatal development?	Accumulation of LAT4 substrates within intestinal epithelial cells and delayed gastrointestinal motility No changes in growth	LAT4 KO in SI does not lead to severe phenotype but alters absorption of substrate AAs and gut transit	Figs 3 and 4
Inducible kidney tubule‐specific LAT4 KO (*LAT4^f/f^ Pax8‐rtTA^+^ LC1^+^*)	Almost 80–90%	Role of LAT4 in the kidney?	Urinary loss of LAT4 and LAT2 substrates under HPD	LAT4 KO in kidney affects reabsorption of all neutral AAs	Figs 6 and 7
Inducible intestine specific LAT4 KO in constitutional global TAT1 KO (*TAT1^–/–^ LAT4^f/f^ Villin‐CreER^T2+^*)	Almost 100% LAT4 KO in SI and global TAT1 KO	Are the uniporters LAT4 and TAT1 together indispensable for net basolateral amino acid transport in SI?	TAT1 KO display accumulation of Trp (TAT1 substrate) and Leu (LAT4 substrate) in intestinal epithelial cells whereas TAT1‐LAT4 dKO behaved similar to WT Mild delay in absorption of Trp and Leu immediately after gavage	Yet unknown alternate directional efflux pathways may be activated in the absence of both uniporters	Fig. 5
Inducible global LAT4 KO in constitutional global TAT1 KO (*TAT1^–/–^ LAT4^f/f^ Rosa‐CreER^T2+^*)	LAT4 KO almost 100% in SI and 60% in kidney, 100% global TAT1 KO	Are the uniporters LAT4 and TAT1 together indispensable for net basolateral amino acid transport in kidney?	Synergistic urinary loss of LAT4, TAT1 and LAT2 substrates (almost all AAs) even under NPD	LAT4 and TAT1 play essential role in kidney reabsorption of most AAs	Fig. 8

## Additional information

### Competing interests

The authors declare that they have no competing interests.

### Author contributions

All of the experiments were carried out at the University of Zurich in Zurich, Switzerland. AR, NP, LO and FV designed the study and all of the necessary experiments. AR, NP, LO and BH carried out the experiments. AR and FV analysed and interpreted the data. AR and FV wrote and critically revised the article. All authors approved the final version of the article submitted for publication and agree to be held accountable for all aspects of this study. All persons qualifying for authorship are listed as such.

### Funding

This study was supported by the Swiss National Science Foundation grant #31_166430/1.

## Supporting information


**Statistical Summary Document**

## Data Availability

The data that support the findings of this study are available from the corresponding author upon reasonable request.
